# Targeted protein degradation in oncology: novel therapeutic opportunity for solid tumours?

**DOI:** 10.1002/1878-0261.70034

**Published:** 2025-04-23

**Authors:** Noé Herbel, Sophie Postel‐Vinay

**Affiliations:** ^1^ ERC Chromatin Remodelling, DNA Repair and Epigenetics Laboratory, INSERM Unit U981 Gustave Roussy Institute Villejuif France; ^2^ Drug Development Department Gustave Roussy Institute Villejuif France; ^3^ Paris‐Saclay University Paris‐Sud University XI, Faculty of Medicine Le Kremlin‐Bicêtre France; ^4^ University College of London Cancer Institute UK

**Keywords:** E3 ligase, molecular glue, PROTAC, targeted protein degradation, undruggable proteome

## Abstract

Targeted and immune therapies have improved patient outcomes in selected diseases. Still, resistance inevitably occurs, and a significant portion of the proteome remains undruggable due to target localisation, structural or functional constraints. Targeted protein degraders (TPDs) represent a promising strategy to expand druggable targets by redirecting the ubiquitin–proteasome system to selectively degrade proteins of interest (POI). TPDs include proteolysis‐targeting chimeras (PROTACs), which are heterobifunctional molecules that create a ternary complex with the POI and the E3 ligase, and molecular glues (MGs), which are monovalent small molecules that create an interface between an E3 ligase and the POI. Here, we provide a viewpoint on novel therapeutic opportunities offered by TPDs, notably through the targeting of previously undruggable proteins or overcoming some resistance mechanisms. We further present challenges that will need to be addressed in order to optimise clinical development, including dose optimisation, patient selection and drug delivery.

## Graphical abstract

Current anticancer therapies are limited by the occurrence of resistance and undruggability of most proteins. Targeted protein degraders are novel, promising agents that trigger the selective degradation of previously undruggable proteins through the recruitment of the ubiquitin–proteasome machinery. Their mechanism of action raises exciting challenges, including patient selection, pharmacokinetics and drug delivery, to be addressed to foster clinical development.

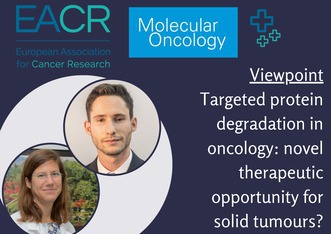

**Abbreviations**
AR: androgen receptor
BRAF: v‐Raf murine sarcoma viral oncogene homologue B
BRD9: bromodomain containing 9
CD3: cluster of differentiation 3
CDK: cyclin‐dependent kinase
EGFR: epidermal growth factor receptor
ER: oestrogen receptor
GSPT1: G1 to S phase transition 1
HDAC: histone deacetylase
HER2: human epidermal growth factor receptor 2
IV: intravenous
KRAS: Kirsten rat sarcoma viral oncogene homologue
MET: mesenchymal–epithelial transition factor
MGs: molecular glues
MoA: mechanism of action
ORR: overall response rate
PARP: poly (ADP‐ribose) polymerase
PD: pharmacodynamic
PK: pharmacokinetic
POI: protein of interest
PROTAC: proteolysis‐targeting chimera
Siglec: sialic acid‐binding immunoglobulin‐type lectin
SMARCA2: SWI/SNF‐related matrix‐associated actin‐dependent regulator of chromatin subfamily A member 2
SMARCA4: SWI/SNF‐related matrix‐associated actin‐dependent regulator of chromatin subfamily A member 4
SMI: small molecule inhibitor
SWI/SNF: SWItch/sucrose non‐fermentable
TKI: tyrosine kinase inhibitor
TPD: targeted protein degrader
WEE1: G2 checkpoint kinase

## 1 Introduction

Since the emergence of precision medicine in the early 2000s, the understanding of cancer molecular alterations has become critical to classify tumours and orient therapeutic decision [1]. While the advent of small molecule inhibitors (SMI), such as tyrosine kinase inhibitors (TKIs), has enabled the improvement of patient outcomes in appropriately molecularly selected diseases, resistance unavoidably emerges, ultimately limiting therapeutic efficacy [2]. Also, SMIs require the presence of a structured and targetable catalytic pocket, making 85% of the proteome ‘undruggable’, including proteins that play a critical role in oncogenesis, such as transcription factors or disordered proteins [3, 4]. Besides SMIs, monoclonal antibodies represent the second largest class of drugs that have brought significant patient benefit. Still, the latter cannot penetrate within the cell and accordingly target transmembrane proteins to perform one of its intended functions: (a) block receptor signalling as naked antibodies (e.g. HER2 with trastuzumab), (b) deliver cytotoxic payloads conjugated to the antibody (antibody‐drug conjugates, e.g. trastuzumab‐deruxtecan) or (c) induce antibody dependent cellular cytotoxicity through the antibody Fc fragment. Bi‐ or tri‐specific antibodies can further either target multiple proteins on the same tumour cell (EGFRxMET with amivantamab) or engage immune cells (e.g. T‐cell engagers). More recent approaches, such as cell therapy or vaccines, also target surface proteins or neoepitopes, thereby leaving a wide number of intracellular oncogenic proteins undruggable.

In this context, targeted protein degradation offers novel promises to both enlarge the spectrum of actionable proteins and also overcome acquired resistance to other therapy modalities.

## 2 Targeted protein degraders: mechanism of action

The ubiquitin–proteasome pathway is the main cellular pathway used to degrade dysfunctional proteins and maintain protein homeostasis. Targeted protein degraders (TPD) exploit this machinery to selectively degrade proteins of interest (POI) by concomitantly binding to the POI and recruiting an E3 ubiquitin ligase, ultimately leading to the poly‐ubiquitination and subsequent POI degradation. Two classes of TPD are currently used in cancer care: proteolysis‐targeting chimera (PROTAC) and molecular glues (MGs). PROTACs are large, heterobifunctional molecules, comprising a POI binding site, a linker and an E3 ligase binding site. The forced creation of this E3 ligase‐PROTAC‐POI ternary complex leads to the ubiquitination and degradation of the POI. By contrast, MGs are monovalent, small molecules that create a binding interface between a recruited E3 ligase and POI. Because the mechanism of action (MoA) of TPDs relies on POI degradation and structural affinities rather than on inhibiting an enzymatic activity, resistance is less likely to occur than with SMI, and as a result, the druggable proteome is significantly enlarged.

## 3 Targets amenable to protein degradation

**Fig. 1:**
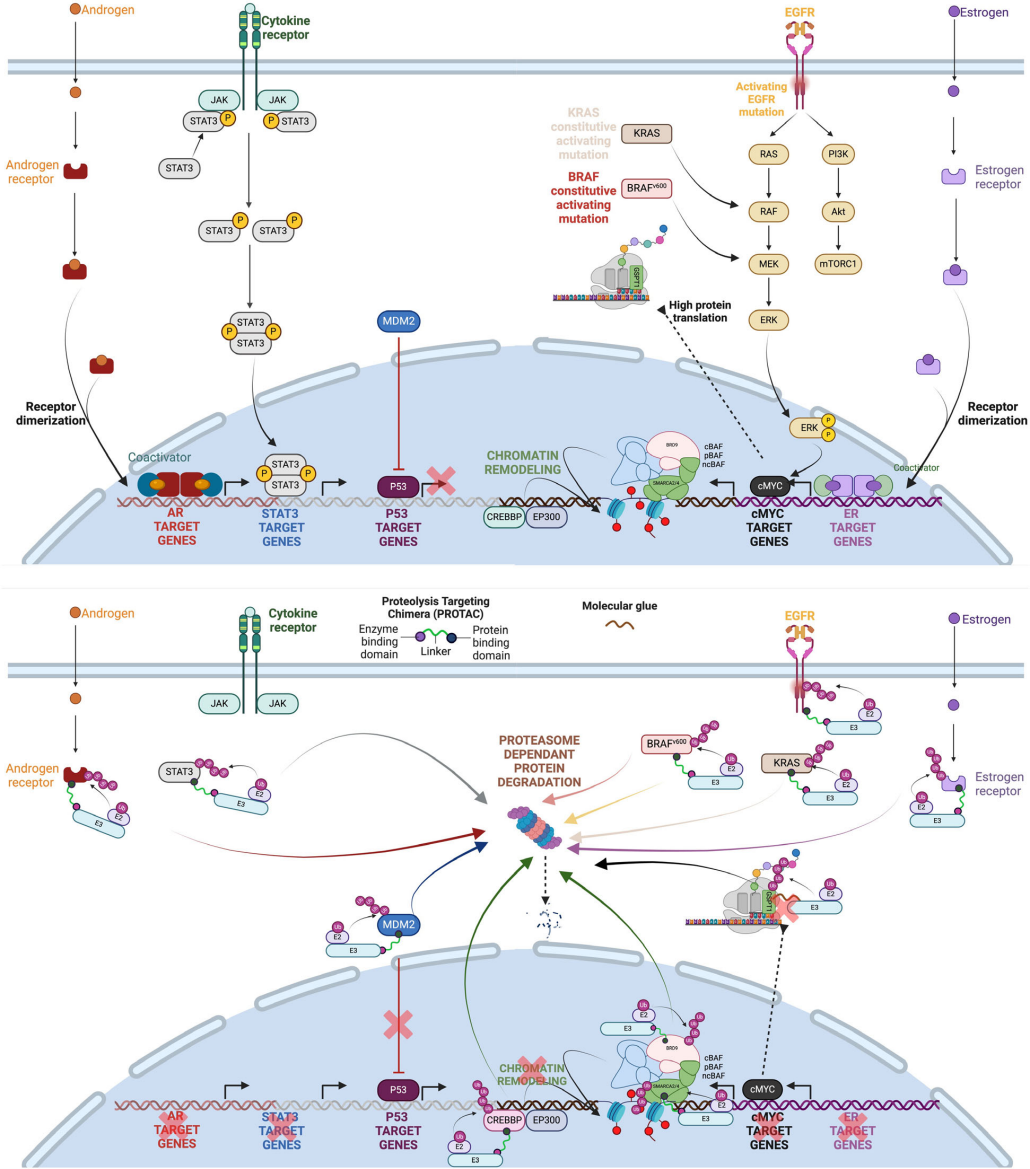
Overview of the main targeted protein degraders in development in solid tumours. Upper panel: overview of the main pathways or proteins of interest targetable by targeted protein degraders (TPD); Lower panel: mechanism of action of TPDs or PROTACs. AR, androgen receptor; BRAFV600, B‐Raf proto‐oncogene, serine/threonine kinase V600 mutation; BRD9, bromodomain containing 9; CREBBP, CREB‐binding protein; ER, oestrogen receptor; GSPT1, G1 To S phase transition 1; JAK, janus kinase; KRAS, Kirsten rat sarcoma viral oncogene homologue; MDM2, mouse double minute 2 homologue; MYC, myelocytomatosis oncogene; STAT3, signal transducer and activator of transcription 3. Figure made with Biorender, https://BioRender.com/h44a056.

The MoA of TPD enables targeting POI with various functions, structures and cellular localisations (Fig. 1). Like SMIs, degraders can target cytoplasmic enzymes, including kinases (BRAF [5] and KRAS [6]) or nonkinase proteins (e.g. inhibitor of apoptosis proteins [7]), or nuclear proteins (e.g. CDK9 [8], WEE1 [9] kinases, HDAC [10] or PARP [11]). Similar to antibodies, degraders can also target membrane proteins (e.g. EGFR [12] or oestrogen receptor, ER [13]). Beyond these, degraders now enable targeting additional POI which either contain several catalytic domains whose isolated inhibition would be insufficient (e.g. SMARCA2 [14] and BRD9 [15]) or whose activity relies on cooperative interactions, structural rearrangements and conformational changes (GSPT1 [16, 17]). Finally, TPD can also target POI from the tumour microenvironment which play an essential role in tumour growth, such as the glycoimmune checkpoints Siglec‐7 and Siglec‐9 [18].

## 4 Drug discovery methods for TPD

Two main approaches exist to develop TPDs: the ‘supervised’ and the ‘agnostic’ method. The supervised approach is aimed at developing a degrader targeting a known POI, mainly in the form of PROTACs. To do so, a known binder of the target is needed. The latter can either leverage existing binders—as for the ARV‐110 androgen receptor (AR) that is made of the AR antagonist bicalutamide fused to the E3 ligase domain of thalidomide—or be developed using methods, such as phage‐display or DNA‐encoded libraries, which allow systematic screening of potential binders to a POI. The agnostic approach is based on high‐throughput screening of TPD libraries, usually composed of small molecule MGs. In this latter case, cell survival is the initial readout; once a cell death‐inducing TPD has been identified, its target is secondarily characterised, most frequently using mass spectrometry.

## 5 Pharmacodynamic (PD)/pharmacokinetic (PK) characteristics

**Fig. 2:**
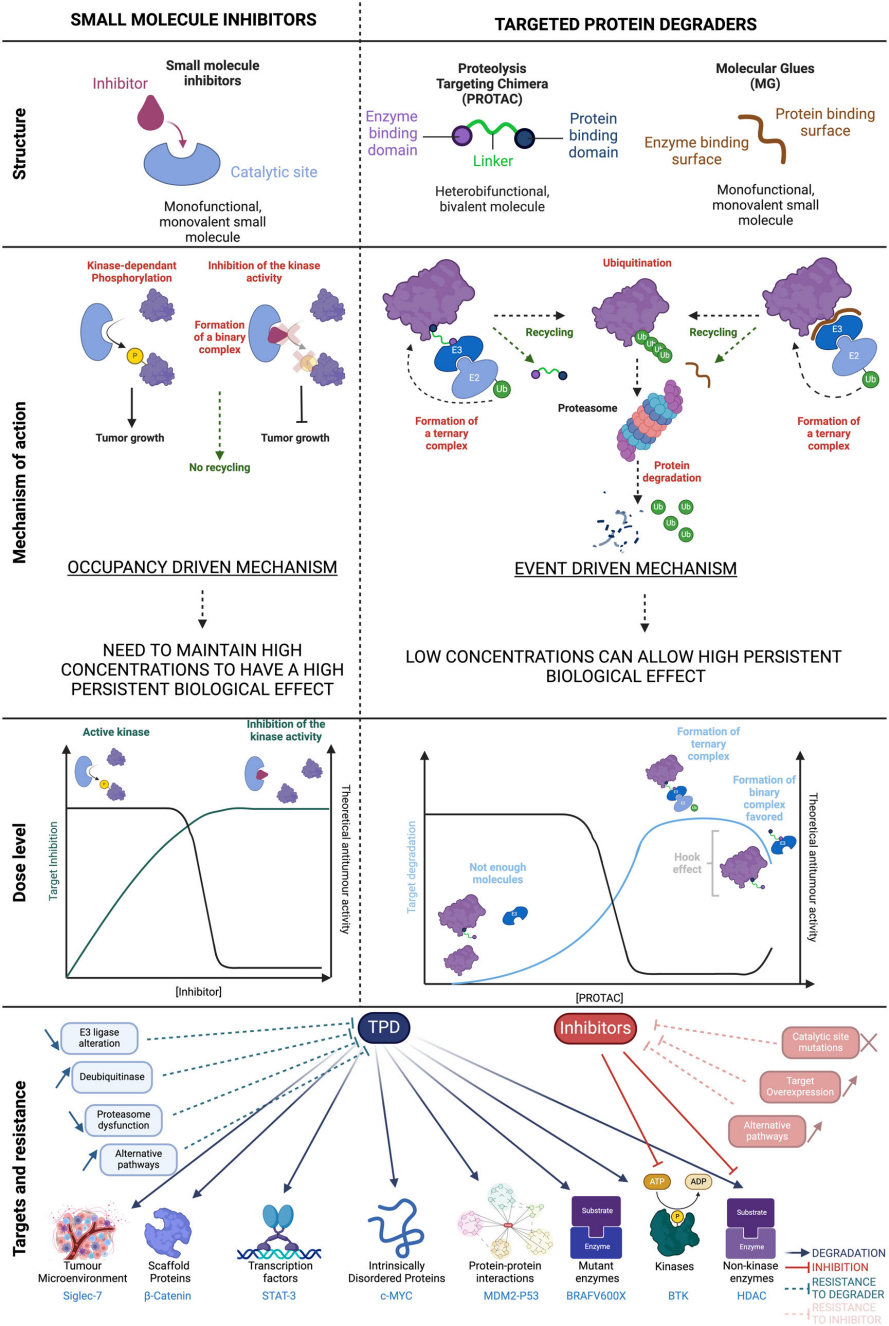
Main differences between targeted protein degraders and small molecule inhibitors (SMIs). Comparison of the structure, mechanism of action, dose–response profile, druggable targets and mechanism of resistance of small molecule inhibitors *versus* protein degraders. BRAFV600, B‐Raf proto‐oncogene, serine/threonine kinase V600 mutation; BTK, Bruton's tyrosine kinase; HDAC, histone deacetylase; MDM2, mouse double minute 2 homologue; MYC, myelocytomatosis oncogene; Siglec, sialic acid‐binding immunoglobulin‐type lectins; STAT3, signal transducer and activator of transcription 3. Figure made with Biorender, https://BioRender.com/u00c306.

All TPDs work by forming a ternary complex between an E3 ligase and the POI, leading to its ubiquitination and subsequent degradation (Fig. 2). Hence, an optimal formation of that ternary complex leads to optimal degradation; once one POI is degraded, the TPD can engage with new POIs, allowing a sustained and prolonged activity. Therefore, both PROTAC and MGs can be active at very low concentrations (as opposed to SMI which require high concentrations and target occupancy to lead to clinical efficacy). For instance, in the phase I dose escalation study of the ER degrader vepdegestrant (Arvinas), near complete ER degradation was observed even at the lowest doses [13]. However, PK characteristics of PROTACs and MGs differ at high concentrations. For PROTACs, activity will initially increase with dose and then decrease with higher concentrations following a bell‐shaped curve according to the Hook effect (i.e. the predominance of binary complexes between the PROTAC and POI or E3 ligase, rather than ternary complexes) [19]. Such phenomena will not occur with MGs, which can only interact with the POI once they have bound the E3 ligase. This difference may be interesting when selecting the most relevant TPD type to be developed.

## 6 Degraders in the clinic: current perspectives

### 6.1 Increasing therapeutic window and overcoming resistance to SMIs

Degraders finally may display increased potency and higher selectivity as compared to their cognate SMI, as their MoA is event‐driven rather than occupancy‐driven. Thanks to this, efficacy can be reached at lower dose levels, with intermittent schedules—thereby limiting off‐target effects—or be observed in SMI‐resistant patients. For example, while ER‐targeting therapies with clinical efficacy already exist (e.g. fulvestrant), vepdegestrant showed a better efficacy in preclinical models. Most importantly, remarkable clinical efficacy was observed in patients who were pretreated and progressed under ER and CDK4/6 inhibitors, with an overall response rate (ORR) of 41.9% in combination with the CDK4/6 inhibitor palbociclib. A 47.1 ORR was further observed in patients with *ESR1* mutations [13]—an alteration that is associated with resistance to ER‐targeting therapies, leading to the FDA approval of this PROTAC in 2024 for patients with ER+/HER− advanced breast cancer [20]. Similarly, the AR PROTAC luxedegalutamide (ARV‐766) showed clinical efficacy in patients with metastatic castration‐resistant prostate cancer with acquired resistance and AR binding domain mutation [21]. Beyond hormone receptors, PROTACs also show promise in targeting oncogenes for which SMI are already approved, as illustrated by the efficacy and limited toxicity of CFT1946, which specifically targets BRAF V600 mutant, sparing BRAF wild‐type [22].

### 6.2 Drugging the undruggable

TPD allows targeting a new spectrum of proteins, including scaffolding proteins, proteins with ill‐defined or flexible structures, and proteins with cooperative or iterative mechanisms of action. For example, SMARCA2 is a nuclear subunit of the SWI/SNF complex, whose ATPase, helicase, scaffolding and bromodomain functions are important in cancer. Disabling SMARCA2 is synthetic lethal with the loss of its paralog SMARCA4, which is frequently mutated in multiple solid tumours, including nonsmall cell lung cancer, bladder cancer and small cell carcinoma of the ovary. Accordingly, PRT3789—a SMARCA2‐selective degrader—was recently evaluated in a Phase 1 trial, with responses observed in some SMARCA4‐mutant tumours, thereby demonstrating the proof‐of‐concept utility of this approach [23]. Similarly, MGs have been used for decades in haematological malignancies in the form of ‐imids, and these are only now being developed in solid tumours. For example, MRT‐2359 is a GSPT1‐TPD currently evaluated in phase 1 trials to target Myc‐amplified tumours, a context where all previous SMI—such as bromodomain extra‐terminal inhibitors—have failed. Myc‐addicted tumours, which may represent up to 70% of all human cancers [24], are indeed highly dependent on protein synthesis, for which GSPT1 is a rate‐limiting factor of the translation termination step [17].

## 7 Challenges to be addressed

TPD represents a new class of drugs in solid tumour oncology that offers novel advantageous characteristics and represents a promising extension of the current anticancer therapeutic armamentarium, with efficacy observed in multiple recent clinical trials. Still, several challenges remain to be addressed. Firstly, defining the optimal recommended dose will be key, as high doses can be less efficacious than lower doses for PROTACs. Thorough PK/PD exploration in early clinical trials is therefore crucial, and randomised dose‐finding trials may be recommended once dose escalation has been completed. Secondly, patient selection is more complex than for SMI. Indeed, the molecular profile or tumour expression of both the POI and that of the recruited E3 ligase need to be considered for patient selection, whereas only the former is required for SMI. Not all tumours express the same E3 ligases, and primary resistance will occur if the recruited E3 ligase is absent. Similarly, variable expression of the POI (e.g. dynamic induction or epigenetic silencing) or of the E3 ligase will strongly impact the TPD efficacy. Third, drug delivery and distribution need careful optimisation. While PROTACs and MGs can be orally available, their physicochemical properties support that IV administration is better suited for very large molecules, such as PROTACs [25]. Preclinical PK/PD data also suggest that intermittent schedules may be sufficient as the TPD shows prolonged effect even upon decreased plasma concentration [26]. Also, the limited ability of the latter to penetrate within a dense tumour microenvironment, or access to the cell nucleus, may render MGs preferable for tumours with stiff microenvironments or for targeting nuclear POI, such as transcription factors. Finally, selectivity is critical, as low selectivity will cause high toxicity even at low doses, following the event‐driven MoA.

In conclusion, TPD represents a promising avenue towards cancer treatment which is still in its infancy in solid tumours: The number of targeted proteins can still exponentially increase, and even if several exciting PK/PD challenges still need to be addressed, recent progress in medicinal chemistry and clinical successes open bright perspectives.

## Conflict of interest

NH declares no conflict of interest. SP‐V is PI of studies sponsored by Amgen, AstraZeneca, Oxford Biotherapeutics, Beigene, MSD, Roche, Novartis, Clever Therapeutics, OSE Immunotherapeutics, Dragonfly therapeutics and GlaxoSmithKline (Institutional funding). SP‐V research team has received research funding from AstraZeneca, AMGEN and Hoffman LaRoche (IMCore) for research projects unrelated to this manuscript (Institutional funding). SP‐V has served in Advisory Boards for AMGEN, Daiichi‐Sankyo and Epics Therapeutics, and has received consulting fees from Epics Therapeutics.

## Author contributions

NH and SP‐V conceptualised the viewpoint, conducted the literature review and wrote the manuscript.
